# Vitamin C Alleviates the Risk of Gestational Diabetes Mellitus Associated With Exposure to Metals

**DOI:** 10.1155/2024/1298122

**Published:** 2024-07-13

**Authors:** Ying Wang, Weiwei Wu, Ping Zhang, Xi Chen, Yongliang Feng, Hailan Yang, Lan Jin, Huang Huang, Xiaoming Shi, Suping Wang, Yawei Zhang

**Affiliations:** ^1^ Department of Epidemiology Shanxi Medical University School of Public Health, Taiyuan, China; ^2^ Center of Clinical Epidemiology and Evidence Based Medicine Shanxi Medical University, Taiyuan, China; ^3^ National Institute of Environmental Health Chinese Center for Disease Control and Prevention, Beijing, China; ^4^ Department of Obstetrics The First Affiliated Hospital Shanxi Medical University, Taiyuan, China; ^5^ Department of Surgery Yale School of Medicine, New Haven, Connecticut, USA; ^6^ Department of Cancer Prevention and Control National Cancer Center/National Clinical Research Center for Cancer Hospital Chinese Academy of Medical Sciences and Peking Union Medical College, Beijing, China

**Keywords:** gestational diabetes mellitus, interaction, metals, vitamin C

## Abstract

**Background:** Exposure to heavy metals has been suggested to increase the risk of gestational diabetes mellitus (GDM) through the oxidative stress pathway. The study is aimed at examining whether vitamin C could modify the association between exposure to heavy metals and risk of GDM.

**Methods:** We conducted a case-control study in Taiyuan, China, with 776 GDM cases and 776 controls. Data on vitamin C intake from diet and supplements were collected through questionnaires. Concentrations of metals in participants' blood were measured using inductively coupled plasma-mass spectrometry (ICP-MS). Unconditional logistic regression models were applied to estimate effect modification of vitamin C on the association between heavy metals and GDM.

**Results:** Women with higher blood levels of mercury (Hg) (odds ratio (OR) = 2.36, 95% confidence interval (CI): 1.43, 3.92 and 2.04, 95% CI: 1.20, 3.46 for the second and third vs. the first tertile) and arsenic (As) (OR = 2.46, 95% CI: 1.37, 4.43 and 2.16, 95% CI: 1.12, 4.17 for the second and third vs. the first tertile) exposure were associated with increased risk of GDM among women without vitamin C supplement use and having dietary vitamin C intake < 85 mg/day. We found no significant association with metals among women who took vitamin C supplements and/or dietary vitamin C ≥ 85 mg/day. Significant interactions were observed between vitamin C and exposures to metals (i.e., Hg and As) on the risk of GDM (*P*_interaction_ = 0.048 and 0.045, respectively).

**Conclusions:** Our study, for the first time, suggests that vitamin C supplement use or higher dietary vitamin C intake during preconception and early pregnancy could alleviate the risk of GDM associated with exposure to As and Hg. The results warrant further investigation.

Summary

• First, the study examined whether vitamin C modifies heavy metals and GDM relationship.
• The risk of GDM associated with exposure to As and Hg could be modified.
• Vitamin C supplements and higher dietary vitamin C intake could alleviate the risk.

## 1. Introduction

Gestational diabetes mellitus (GDM) arises when a pregnant woman's pancreatic function cannot meet the growing insulin demands. The prevalence of GDM in China has increased dramatically from 2.3% in 1999 to 9.3% in 2015 [1]. GDM has been considered as a temporary circumstance of Type 2 diabetes mellitus (T2DM) [2]. Hyperglycemia during pregnancy has not only been associated with adverse birth outcomes but has also been linked to long-term severe health outcomes for both mothers and their children, such as metabolic syndrome, cardiovascular diseases in mothers and obesity, and T2DM in their offspring [3, 4].

Although the etiology and pathogenesis of GDM are not well understood, epidemiological and experimental studies have demonstrated a relationship between GDM and oxidative stress [5, 6]. Oxidative stress refers to an imbalance between oxidants and antioxidants, associated with an elevation of reactive oxygen species (ROS) that threaten cell survival [7]. Studies have shown that oxidative stress can influence the function of islet *β*-cells, damaging insulin secretion and glucose metabolism in both *in vitro* and *in vivo* experiments [8–10]. Studies have also found that oxidative stress causes abnormal transcription of the glucose transporter 4 (GLUT4) and subsequently reduces glucose transport [11, 12]. Increased oxygen radical damage biomarkers, including DNA damage markers and lipid peroxidation products, have been found to be associated with GDM risk [13, 14].

Vitamin C, also called ascorbic acid, is a water-soluble vitamin and an important antioxidant. It can scavenge ROS and nitric oxide to protect DNA, proteins, and lipids from damage [15]. Animal studies have shown that vitamin C has a protective role against the risk of diabetes mellitus by preventing abnormal insulin secretion due to oxidative stress [16, 17]. Accumulating evidences have suggested that vitamin C supplements can improve insulin action in individuals with T2DM [18–21]. The relationship between vitamin C and GDM has thus far been inconclusive [22–26].

Recent studies have shown that heavy metals can accumulate in pancreatic tissue, leading to insulin resistance [27, 28]. A growing number of epidemiological studies have suggested that women with higher levels of heavy metal (blood or hair) exposure may promote the development of GDM [27, 29–32]. Heavy metals may impair glucose tolerance due to functional impairments in insulin secretion among *β*-cells, leading to GDM [33, 34]. Our previous study found that women with higher levels of heavy metals blood exposure were associated with increased risk of GDM, and this increased risk is mainly driven by Hg and, to a lesser extent, by Ni, Pb, and As [35]. One of the potential mechanisms underlying this association is that the function and survival of islet *β*-cells were affected by heavy metals through oxidative stress pathways. Given the role of vitamin C on antioxidative stress, we hypothesize that increased intake of vitamin C may alleviate the harmful impact of metals on GDM risk. To test this hypothesis, we analyzed data from the Taiyuan birth cohort study conducted in 2012–2016 [35–37].

## 2. Materials and Methods

### 2.1. Study Population

The study population has been described earlier [35]. In brief, 776 GDM women and 776 non-GDM controls were identified from the Taiyuan birth cohort with the following inclusion criteria: (a) had a singleton live birth without birth defects and (b) donated blood samples. GDM cases were defined as those who had fasting blood levels of more than 5.1 mmol/L, or 1-h blood glucose>10.0 mmol/L, or 2-h blood glucose>8.5 mmol/L during the gestational Weeks 24–28. Controls were randomly selected and were frequency matched to cases by age (± 2 years), conception time (± 3 months) and place of residence. Information on demographics, reproductive and medical history, lifestyle factors, nutrient supplements, and diet was collected through in-person interviews. All study procedures were approved by the Ethics Committee at the Shanxi Medical University.

### 2.2. Vitamin C Supplements and Dietary Vitamin C Intake

As the diagnosis of GDM took place during gestational Weeks 24–28, vitamin C supplement use and dietary vitamin C intake during preconception (the 12 months before pregnancy) and the first trimester (1–13 weeks) were considered. Vitamin C supplement users were defined as those who took vitamin C supplements alone or vitamin C containing multivitamins during the preconception period and/or during early pregnancy. Dietary information was collected through a validated 33-item food frequency questionnaire (FFQ) [36, 38]. The 33 food items include cereals (rice, wheat flour, and coarse food grain), meats (pork, beef, mutton, poultry, freshwater fish, marine fish, shrimp, crab, and shell fish), dairy (milk, milk powder, and yogurt), eggs, bean products (soybean milk, tofu, kidney beans, and cowpeas), green leafy vegetables, other vegetables (cabbages, carrots, tomatoes, eggplants, potatoes, mushrooms, peppers, bamboo shoots, agarics, and garlic), alga (nori and kelp), pickles, nuts, and fruits. Participants need to report the frequency and consumption of standard portion size for each food, and then, we converted data into grams per day. Dietary vitamin C intake was estimated based on the frequency and amount of each consumed food item according to the Chinese Standard Tables of Food Consumption for each time period [39]. As the recommended dietary allowance for vitamin C intake during pregnancy is 85 mg/day according to the Institute of Medicine Panel on Dietary Antioxidants and Related Compounds, we categorized dietary vitamin C intake into < 85 mg/day and ≥ 85 mg/day [40]. Finally, we combined vitamin C supplement use and dietary vitamin C intake and then categorized them into three groups: (a) vitamin C supplement nonusers and dietary vitamin C intake < 85 mg/day (nonusers and < 85 mg/day), (b) vitamin C supplement nonusers and dietary vitamin C intake ≥ 85 mg/day or vitamin C supplement users and dietary vitamin C intake < 85 mg/day (nonusers and ≥ 85 mg/day or users and < 85 mg/day), and (c) vitamin C supplement users and dietary vitamin C intake ≥ 85 mg/day (users and ≥ 85 mg/day).

### 2.3. Analysis of Metals

Maternal blood samples were collected and stored in EDTA tubes at −80°C for further analysis. Blood concentrations of heavy metals (Ni, As, Cd, Sb, Tl, Hg, and Pb) were measured using inductively coupled plasma-mass spectrometry (ICP-MS). Briefly, 300 *μ*L blood samples were mixed with BV-III HNO_3_ (200 *μ*L) and 30% H_2_O_2_ (100 *μ*L) and digested using the Mars 5 microwave accelerated reaction system. Digested samples were diluted with deionized water to a nitric acid concentration of 10%. Each batch (20 samples) contained one positive sample (the Seronorm™ trace elements whole blood L-2 [LOT 1003192]) and one negative sample (deionized water) for quality control purposes. A total of 78 duplicated samples were also included with intra-assay and interassay coefficient of variances (CVs) ranging from 0.23% to 2.53%. Each batch of samples included both cases and controls. Laboratory personnel were blinded to GDM status.

### 2.4. Statistical Analysis

We used unconditional logistic regression models to calculate the odds ratios (ORs) and 95% confidence intervals (CIs). According to our previous study, if metals had ≥ 80% of detectable measurements, those metals were classified into tertiles based on their distributions among controls. For metals with < 80% of detectable measurements, the measurements below the limit of detection (LOD) were categorized as the reference group, while the measurements above the LOD were divided into two groups based on the median of the control group [35]. Trend analysis was conducted using ordinal variables (1, 2, and 3) in the model. To evaluate the potential effect modification of vitamin C supplement use and dietary vitamin C intake, we performed stratified analyses by vitamin C supplement use (nonusers and users) and dietary vitamin C intake (< 85 mg/day and ≥ 85 mg/day), respectively, and then by vitamin C supplement use and dietary vitamin C intake combined groups (nonusers and < 85 mg/day, nonusers and ≥ 85 mg/day or users and < 85 mg/day, and users and ≥ 85 mg/day). Statistical tests for interactions were conducted through cross-product terms in the multivariate logistic regression models. Potential confounding variables included parity, month of conception, family history of diabetes, gestational weight gain, physical activities, and prepregnancy BMI according to the previous study [35]. *p* < 0.05 was considered as statistically significant. All analyses were performed using SAS 9.4 version.

## 3. Results

As shown in Table 1, no significant associations were observed between GDM and vitamin C supplement use and dietary vitamin C intake.

As we reported previously [35], women with higher levels of As (OR = 1.49, 95% CI: 1.11, 2.01 for the second tertile vs. the first tertile) and Hg (OR = 1.43, 95% CI: 1.09, 1.88 for the third tertile vs. the first tertile) blood exposure were associated with increased risk of GDM. After stratification by vitamin C supplement use (Table 2), among nonusers, we found that women with higher levels of As blood exposure (OR = 1.53, 95% CI: 1.13, 2.06 and 1.43, 95% CI: 1.01, 2.03 for the second and third tertiles vs. the first tertile, *P*_trend_ = 0.134) was associated with increased risk of GDM, whereas there was no significant association between As and GDM among vitamin C supplement users. The interaction between As and vitamin C supplement use on the risk of GDM was statistically significant (*P*_interaction_ = 0.047). No significant associations and interactions were observed for other metals.

After stratification by dietary vitamin C intake (Table 3), higher levels of Hg blood exposure (OR = 2.42, 95% CI: 1.48, 3.95 and 2.20, 95% CI: 1.30, 3.70 for the second and third tertiles vs. the first tertile, *P*_trend_ = 0.008) was associated with elevated risk of GDM among women with dietary vitamin C intake < 85 mg/day, but no significant association between Hg and GDM was observed among women with dietary vitamin C intake ≥ 85 mg/day. The difference in the association between Hg and the risk of GDM by dietary vitamin C intake was statistically significant (*P*_interaction_ = 0.044). No significant associations and interactions were observed for other metals.

After consideration of both vitamin C supplement use and dietary vitamin C intake (Table 4), we observed that higher levels of Hg (OR = 2.36, 95% CI: 1.43, 3.92 and 2.04, 95% CI: 1.20, 3.46 for the second and third vs. the first tertile, *P*_trend_ = 0.021) and As (OR = 2.46, 95% CI: 1.37, 4.43 and 2.16, 95% CI: 1.12, 4.17 for the second and third vs. the first tertile, *P*_trend_ = 0.065) blood exposure were associated with increased risk of GDM among women without vitamin C supplement use and having dietary vitamin C intake < 85 mg/day. We found no significant associations with metals among other two groups (nonusers and ≥ 85 mg/day or users and < 85 mg/day, users and ≥ 85 mg/day). The observed associations of GDM with Hg and As among the different groups were statistically significant (*P*_interaction_ = 0.048 and 0.045, respectively).

## 4. Discussion

Our study, for the first time, evaluated the effect modification of vitamin C supplement use and dietary vitamin C intake during preconception and early pregnancy on the association between exposure to metals and GDM. We found that vitamin C supplement use and high dietary vitamin C intake could alleviate the risk of GDM associated with maternal blood exposure to As and Hg.

Several studies have examined the association between vitamin C and GDM risk and have presented inconsistent results [22–26]. A study from Poland with 29 GDM cases found that dietary vitamin C was associated with GDM by univariate analysis [23]. However, a study from Iran involving 40 GDM cases found that there were no statistically significant associations between serum vitamin C levels and GDM [25]. Meanwhile, a cross-sectional research in India including 25 GDM cases observed that the GDM group had lower serum vitamin C levels [22]. A study in the United States with 30 GDM cases found that women with low dietary vitamin C intake (< 70 mg/day) had a twofold risk of GDM compared with higher intake, adjusting for household income, family history of T2DM, parity, prepregnancy adiposity, race, and maternal age [26]. Additionally, a longitudinal cohort study from China with 344 GDM cases found that women with higher dietary vitamin C intake (≥ 200 mg/day) had a lower risk of GDM than those with normal dietary vitamin C intake (115–200 mg/day); however, no association was found between total vitamin C intake (sum of dietary vitamin C and vitamin C from supplements) and GDM risk [24]. Our study did not find significant associations between dietary vitamin C intake or vitamin C supplements and the risk of GDM. A possible explanation for the variety of these conclusions is that some studies used dietary vitamin C intake while others used serum vitamin C levels. Although some studies have shown that higher vitamin C could favorably influence glucose homeostasis by preventing pancreatic *β*-cells cellular damage from oxidative stress, this influence may be too small to be detected or need higher levels of vitamin C intake.

Both Hg and As are common toxic elements mainly derived from industrial electrical equipment, sewage disposal, and manufacturing disinfectants [41–43]. Studies have demonstrated that exposure to heavy metals could cause the overproduction of ROS and affect the activity of glutathione peroxidase and superoxide dismutase, increasing the risk of oxidative stress [44–48]. Meanwhile, oxidative stress could induce production of inflammatory mediators, gradually impairing *β*-cell function, reducing insulin sensitivity, and increasing insulin resistance, all of which result in GDM [8, 9, 11, 12, 49–51]. Studies have also found that heavy metals could cause abnormal functioning of the liver and cirrhosis, inducing glucose metabolism disorders, gradually resulting in impaired glucose tolerance, causing GDM [52–54]. In our previous report, we found that higher blood levels of As and Hg were associated with GDM, so we speculated that the potential mechanism might be that the function of islet *β*-cells was affected by metals through oxidative stress pathways [35].

Experimental and epidemiological studies have suggested that vitamin C may have beneficial effects in glucose homeostasis by preventing abnormal insulin secretion attributable to pancreatic *β*-cell damage from oxidative stress and by improving glucose transport and insulin sensitivity [16, 17, 21]. Meanwhile, previous researches have demonstrated that vitamin C can not only prevent heavy metal toxicity but also have protective effects against toxicity in the liver [55–64]. Our study results suggesting that vitamin C supplement use and high dietary vitamin C intake reduce the risk of GDM associated with blood exposure to As and Hg seemed to support an antioxidant effect from vitamin C alleviating damage on pancreatic *β*-cells from Hg and As. Future mechanistic studies are needed to understand how vitamin C modifies Hg–GDM and As–GDM relationships.

One of the main strengths of our study was that maternal complications were collected through medical records, which minimized potential disease misclassification. Another strength was a relatively large sample size (776 GDM cases), allowing us to have sufficient statistical power to conduct stratified analyses. Information on vitamin C supplements and dietary vitamin C intake was collected through in-person interviews at delivery, wherein potential recall bias was a concern. As the association between vitamin C and GDM was unclear during the study time period, recall bias, if any, was unlikely to differ between GDM cases and controls, meaning it was unlikely to skew the results in either direction in this study. The concentrations of metals were measured in blood samples collected at delivery, raising a concern about potential reverse causality on Hg–GDM and As–GDM associations. Meanwhile, the study was limited to the sample size in vitamin C user group. Therefore, future researches are needed to validate our study results through large sample size and metal measured before GDM diagnosis.

## 5. Conclusion

Our study supports the hypothesis that vitamin C supplement use and high levels of dietary vitamin C intake during preconception and early pregnancy could alleviate the risk of GDM associated with blood exposure to As and Hg. Future researches are needed to validate our study findings through large sample size and samples taken during pregnancy.

## Figures and Tables

**Table 1:** Associations between vitamin C supplement use, dietary vitamin C intake, and gestational diabetes mellitus.

**Vitamin C**	**N**	**GDM**	**Control**	**OR** ^a^ **(95% CI)**
Vitamin C supplements				
Nonusers	1462	728 (93.81)	734 (94.59)	1.00
Users	90	48 (6.19)	42 (5.41)	1.17 (0.75, 1.83)
Dietary vitamin C intake				
< 85 mg/day	499	248 (31.96)	251 (32.35)	1.00
≥ 85 mg/day	1053	528 (68.04)	525 (67.65)	0.98 (0.78, 1.23)
Vitamin C supplement use and dietary vitamin C intake				
Nonusers and < 85 mg/day	469	231 (29.77)	238 (30.67)	1.00
Nonusers and ≥ 85 mg/day or users and < 85 mg/day	1023	514 (66.24)	509 (65.59)	1.00 (0.80, 1.26)
Users and ≥ 85 mg/day	60	31 (3.99)	29 (3.74)	1.10 (0.63, 1.92)

*Note:* Estimates for vitamin C supplementation were also adjusted for dietary vitamin C intake (< 85 mg/day, ≥ 85 mg/day). Estimates for estimated dietary vitamin C intake were also adjusted for vitamin C supplementation (nonusers, users).

^a^Adjustment: parity, month of conception, family history of diabetes, gestational weight gain, physical activities, and prepregnancy BMI.

**Table 2:** Associations between metals and gestational diabetes mellitus stratified by vitamin C supplement use.

**Metals**	**Vitamin C nonusers (** **n** = 1462**)**			**Vitamin C users (** **n** = 90**)**		
	**GDM**	**Control**	**OR** ^a^ **(95% CI)**	**GDM**	**Control**	**OR** ^a^ **(95% CI)**
Ni						
Low (< 3.00)	281 (38.60)	284 (38.69)	1.00	34 (70.83)	22 (52.38)	1.00
Middle (3.00–11.30)	224 (30.77)	226 (30.79)	1.15 (0.84, 1.56)	8 (16.67)	9 (21.43)	0.26 (0.02, 2.8)
High (≥ 11.30)	223 (30.63)	224 (30.52)	1.05 (0.74, 1.49)	6 (12.50)	11 (26.19)	0.05 (0.01, 1.32)
*P*_trend_			0.540			0.502
As						
Low (< 10.64)	220 (30.22)	247 (33.65)	1.00	14 (29.17)	12 (28.57)	1.00
Middle (10.64–21.12)	272 (37.36)	244 (33.24)	1.53 (1.13, 2.06)	23 (47.91)	13 (30.95)	1.66 (0.25, 10.93)
High (≥ 21.12)	236 (32.42)	243 (33.11)	1.43 (1.01, 2.03)	11 (22.92)	17 (40.48)	0.32 (0.04, 2.61)
*P*_trend_			0.134			0.138
*P*_interaction_						0.047
Cd						
Low (< 0.69)	263 (36.13)	249 (33.92)	1.00	10 (20.83)	10 (23.81)	1.00
Middle (0.69–3.43)	239 (32.83)	233 (31.75)	1.00 (0.75, 1.33)	33 (68.75)	24 (57.14)	3.99 (0.73, 21.8)
High (≥ 3.43)	226 (31.04)	252 (34.33)	0.59 (0.32, 1.08)	5 (10.42)	8 (19.05)	6.02 (0.16, 223.35)
*P*_trend_			0.205			0.191
Sb						
Low (< 0.30)	195 (26.79)	187 (25.48)	1.00	14 (29.17)	7 (16.67)	1.00
Middle (0.30–6.20)	269 (36.95)	269 (36.65)	0.86 (0.62, 1.19)	27 (56.25)	22 (52.38)	0.25 (0.03, 1.97)
High (≥ 6.20)	264 (36.26)	278 (37.87)	1.12 (0.63, 1.99)	7 (14.58)	13 (30.95)	0.47 (0.03, 6.86)
*P*_trend_			0.765			0.179
Tl						
Low (< 0.01)	215 (29.53)	210 (28.61)	1.00	26 (54.17)	19 (45.24)	1.00
Middle (0.01–0.72)	270 (37.09)	263 (35.83)	1.08 (0.78, 1.49)	18 (37.50)	11 (26.19)	6.06 (0.71, 52.09)
High (≥ 0.72)	243 (33.38)	261 (35.56)	0.89 (0.6, 1.33)	4 (8.33)	12 (28.57)	0.58 (0.08, 3.94)
*P*_trend_			0.311			0.914
Hg						
Low (< 0.99)	209 (28.71)	241 (32.83)	1.00	18 (37.50)	18 (42.86)	1.00
Middle (0.99–1.73)	229 (31.46)	246 (33.52)	1.14 (0.86, 1.51)	16 (33.33)	12 (28.57)	0.67 (0.14, 3.24)
High (≥ 1.73)	290 (39.83)	247 (33.65)	1.34 (1.01, 1.77)	14 (29.17)	12 (28.57)	5.73 (0.79, 41.74)
*P*_trend_			0.056			0.192
*P*_interaction_						0.748
Pb						
Low (< 22.54)	221 (30.36)	242 (32.97)	1.00	24 (50.00)	17 (40.48)	1.00
Middle (22.54–33.23)	252 (34.61)	242 (32.97)	1.09 (0.81, 1.46)	16 (33.33)	15 (35.71)	0.38 (0.08, 1.79)
High (≥ 33.23)	255 (35.03)	250 (34.06)	1.12 (0.81, 1.55)	8 (16.67)	10 (23.81)	0.67 (0.08, 5.42)
*P*_trend_			0.527			0.588

^a^Adjustment: parity, month of conception, family history of diabetes, gestational weight gain, physical activities, and prepregnancy BMI.

**Table 3:** Associations between metals and gestational diabetes mellitus stratified by dietary vitamin C intake.

**Metals**	**V** **i** **t** **a** **m** **i** **n** **C** < 85**mg/day (n** = 499**)**			**V** **i** **t** **a** **m** **i** **n** **C** ≥ 85 **mg/day (n** = 1053**)**		
	**GDM**	**Control**	**OR** ^a^ **(95% CI)**	**GDM**	**Control**	**OR** ^a^ **(95% CI)**
Ni						
Low (< 3.00)	133 (53.63)	118 (47.01)	1.00	182 (34.47)	188 (35.81)	1.00
Middle (3.00–11.30)	66 (26.61)	74 (29.48)	1.20 (0.67, 2.16)	166 (31.44)	161 (30.67)	1.11 (0.77, 1.61)
High (≥ 11.30)	49 (19.76)	59 (23.51)	1.14 (0.59, 2.23)	180 (34.09)	176 (33.52)	0.94 (0.62, 1.42)
*P*_trend_			0.632			0.344
As						
Low (< 10.64)	68 (27.42)	90 (35.86)	1.00	166 (31.44)	169 (32.19)	1.00
Middle (10.64–21.12)	101 (40.73)	77 (30.68)	2.36 (1.34, 4.16)	194 (36.74)	180 (34.29)	1.35 (0.95, 1.92)
High (≥ 21.12)	79 (31.85)	84 (33.46)	1.91 (1.01, 3.60)	168 (31.82)	176 (33.52)	1.17 (0.77, 1.79)
*P*_trend_			0.132			0.950
*P*_interaction_						0.200
Cd						
Low (< 0.69)	77 (31.05)	74 (29.48)	1.00	196 (37.12)	185 (35.24)	1.00
Middle (0.69–3.43)	120 (48.39)	106 (42.23)	1.08 (0.65, 1.79)	152 (28.79)	151 (28.76)	0.96 (0.67, 1.35)
High (≥ 3.43)	51 (20.56)	71 (28.29)	0.41 (0.14, 1.23)	180 (34.09)	189 (36.00)	0.69 (0.34, 1.39)
*P*_trend_			0.494			0.359
Sb						
Low (< 0.30)	65 (26.21)	62 (24.70)	1.00	144 (27.27)	132 (25.14)	1.00
Middle (0.30–6.20)	122 (49.19)	109 (43.43)	0.69 (0.39, 1.25)	174 (32.96)	182 (34.67)	0.83 (0.56, 1.23)
High (≥ 6.20)	61 (24.60)	80 (31.87)	0.62 (0.20, 1.91)	210 (39.77)	211 (40.19)	1.28 (0.67, 2.44)
*P*_trend_			0.366			0.986
Tl						
Low (< 0.01)	100 (40.32)	83 (33.07)	1.00	141 (26.70)	146 (27.81)	1.00
Middle (0.01–0.72)	85 (34.28)	100 (39.84)	1.05 (0.59, 1.88)	203 (38.45)	174 (33.14)	1.37 (0.93, 2.01)
High (≥ 0.72)	63 (25.40)	68 (27.09)	1.38 (0.66, 2.89)	184 (34.85)	205 (39.05)	0.88 (0.56, 1.41)
*P*_trend_			0.776			0.594
Hg						
Low (< 0.99)	69 (27.82)	103 (41.04)	1.00	158 (29.92)	156 (29.71)	1.00
Middle (0.99–1.73)	94 (37.90)	74 (29.48)	2.42 (1.48, 3.95)	151 (28.60)	184 (35.05)	0.79 (0.56, 1.11)
High (≥ 1.73)	85 (34.28)	74 (29.48)	2.20 (1.30, 3.70)	219 (41.48)	185 (35.24)	1.10 (0.79, 1.53)
*P*_trend_			0.008			0.536
*P*_interaction_						0.044
Pb						
Low (< 22.54)	85 (34.27)	87 (34.66)	1.00	160 (30.30)	172 (32.76)	1.00
Middle (22.54–33.23)	82 (33.07)	82 (32.67)	0.79 (0.49, 1.30)	186 (35.23)	175 (33.33)	1.18 (0.83, 1.69)
High (≥ 33.23)	81 (32.66)	82 (32.67)	0.90 (0.53, 1.55)	182 (34.47)	178 (33.91)	1.14 (0.77, 1.69)
*P*_trend_			0.856			0.724

^a^Adjustment: parity, month of conception, family history of diabetes, gestational weight gain, physical activities, and prepregnancy BMI.

**Table 4:** Associations between metals and gestational diabetes mellitus stratified by dietary vitamin C intake and vitamin C supplement use.

**Metals**	**Nonusers and < 85 mg/day** **(** **n** = 469**)**			**Nonusers and ≥ 85 mg/day or users and < 85 mg/day (** **n** = 1023**)**			**Users and ≥ 85 mg/day** **(** **n** = 60**)**		
	**GDM**	**Control**	**OR** ^a^ **(95% CI)**	**GDM**	**Control**	**OR** ^a^ **(95% CI)**	**GDM**	**Control**	**OR** ^a^ **(95% CI)**
Ni									
Low (< 3.00)	111 (46.64)	118 (51.08)	1.00	180 (35.36)	178 (34.63)	1.00	15 (51.72)	19 (61.30)	1.00
Middle (3.00–11.30)	73 (30.67)	64 (27.71)	1.26 (0.70, 2.28)	154 (30.26)	162 (31.52)	1.11 (0.76, 1.61)	8 (27.59)	6 (19.35)	0.06 (< 0.01, 7.40)
High (≥ 11.30)	54 (22.69)	49 (21.21)	1.33 (0.68, 2.63)	175 (34.38)	174 (33.85)	0.91 (0.60, 1.37)	6 (20.69)	6 (19.35)	0.31 (0.01, 69.34)
*P*_trend_			0.925			0.727			0.522
As									
Low (< 10.64)	85 (35.72)	63 (27.27)	1.00	167 (32.81)	162 (31.52)	1.00	7 (24.14)	9 (29.03)	1.00
Middle (10.64–21.12)	74 (31.09)	93 (40.26)	2.46 (1.37, 4.43)	173 (33.99)	187 (36.38)	1.30 (0.91, 1.85)	10 (34.48)	15 (48.39)	3.25 (0.22, 47.97)
High (≥ 21.12)	79 (33.19)	75 (32.47)	2.16 (1.12, 4.17)	169 (33.20)	165 (32.10)	1.15 (0.75, 1.76)	12 (41.38)	7 (22.58)	0.41 (0.02, 9.96)
*P*_trend_			0.065			0.879			0.276
*P*_interaction_									0.045
Cd									
Low (< 0.69)	72 (30.25)	72 (31.17)	1.00	179 (35.17)	196 (38.13)	1.00	8 (27.59)	5 (16.13)	1.00
Middle (0.69–3.43)	98 (41.18)	109 (47.19)	1.14 (0.68, 1.92)	143 (28.09)	141 (27.43)	0.88 (0.62, 1.26)	16 (55.17)	22 (70.97)	3.51 (0.25, 49.36)
High (≥ 3.43)	68 (28.57)	50 (21.64)	0.40 (0.13, 1.22)	187 (36.74)	177 (34.44)	0.63 (0.31, 1.28)	5 (17.24)	4 (12.90)	37.37 (0.02, > 999.99)
*P*_trend_			0.602			0.137			0.081
Sb									
Low (< 0.30)	59 (24.79)	61 (26.41)	1.00	131 (25.74)	138 (26.85)	1.00	4 (13.79)	10 (32.26)	1.00
Middle (0.30–6.20)	103 (43.28)	110 (47.62)	0.65 (0.36, 1.19)	172 (33.79)	171 (33.27)	0.94 (0.63, 1.41)	16 (55.17)	15 (48.39)	0.07 (0.01, 1.58)
High (≥ 6.20)	76 (31.93)	60 (25.97)	0.62 (0.20, 1.95)	206 (40.47)	205 (39.88)	1.37 (0.70, 2.68)	9 (31.04)	6 (19.35)	0.10 (0.01, 4.21)
*P*_trend_			0.278			0.625			0.214
Tl									
Low (< 0.01)	77 (32.35)	88 (38.09)	1.00	139 (27.31)	139 (27.04)	1.00	13 (44.83)	14 (45.16)	1.00
Middle (0.01–0.72)	95 (39.92)	82 (35.50)	1.03 (0.57, 1.87)	173 (33.99)	191 (37.16)	1.20 (0.81, 1.78)	6 (20.69)	15 (48.39)	9.85 (0.16, 591.33)
High (≥ 0.72)	66 (27.73)	61 (26.41)	1.31 (0.63, 2.76)	197 (38.70)	184 (35.80)	0.86 (0.53, 1.39)	10 (34.48)	2 (6.45)	0.17 (0.01, 3.52)
*P*_trend_			0.737			0.545			0.320
Hg									
Low (< 0.99)	97 (40.76)	66 (28.57)	1.00	150 (29.47)	146 (28.40)	1.00	12 (41.38)	15 (48.39)	1.00
Middle (0.99–1.73)	71 (29.83)	87 (37.66)	2.36 (1.43, 3.92)	178 (34.97)	149 (28.99)	0.85 (0.60, 1.21)	9 (31.03)	9 (29.03)	0.10 (0.01, 1.40)
High (≥ 1.73)	70 (29.41)	78 (33.77)	2.04 (1.20, 3.46)	181 (35.56)	219 (42.61)	1.17 (0.84, 1.64)	8 (27.59)	7 (22.58)	0.29 (0.01, 12.51)
*P*_trend_			0.021			0.313			0.794
*P*_interaction_									0.048
Pb									
Low (< 22.54)	82 (34.46)	78 (33.77)	1.00	165 (32.42)	150 (29.18)	1.00	12 (41.38)	17 (54.84)	1.00
Middle (22.54–33.23)	77 (32.35)	76 (32.90)	0.80 (0.48, 1.32)	170 (33.40)	182 (35.41)	1.24 (0.86, 1.78)	10 (34.48)	10 (32.26)	0.30 (0.02, 4.13)
High (≥ 33.23)	79 (33.19)	77 (33.33)	0.90 (0.52, 1.56)	174 (34.18)	182 (35.41)	1.24 (0.83, 1.85)	7 (24.14)	4 (12.90)	0.03 (< 0.01, 3.19)
*P*_trend_			0.802			0.407			0.135

^a^Adjustment: parity, month of conception, family history of diabetes, gestational weight gain, physical activities, and prepregnancy BMI.

## Data Availability

The data used to support the findings of this study are included within the article.
